# Biomarkers predicting postoperative adverse outcomes in children with congenital heart disease: a systematic review and meta-analysis

**DOI:** 10.3389/fped.2025.1508329

**Published:** 2025-01-17

**Authors:** Shifan Zhou, Lu Liu, Xiaochuang Jin, Daniel Dorikun, Songfeng Ma

**Affiliations:** ^1^ Pediatric Cardiothoracic Surgery, First Affiliated Hospital of Xinjiang Medical University, Urumqi, China; ^2^ College of Pediatrics, Xinjiang Medical University, Urumqi, China; ^3^ Hematology Department, First Affiliated Hospital of Xinjiang Medical University, Urumqi, China

**Keywords:** congenital heart disease (CHD), biomarkers, postoperative outcomes, metaanalysis, pediatric cardiac surgery

## Abstract

**Objective:**

To statistically analyze biomarkers predicting postoperative outcomes in children with congenital heart disease (CHD).

**Methods:**

PubMed, Embase, Cochrane Library, and Web of Science were performed to search up to February 2024. The measured outcomes were biomarkers, mortality, length of hospital stay, complication rates, and infection rates. Adults with CHD were excluded. Standard deviation or odds ratio (OR) with 95% confidence interval (95% CI) were extracted. A random-effects model synthesized SMDs or ORs with 95% CIs. Sensitivity analysis investigated heterogeneity, and Egger's test assessed publication bias.

**Results:**

Seventeen eligible articles were included, the biomarkers involved include serum lactate, NT-Pro BNP, PaO2, serum creatinine, C1-INH activity, ST2, serum chloride concentration, GH, glycemia, cTOI, NLR, serum albumin, and glucose levels, with 2,888 patients who underwent surgery(modified Norwood procedure, arterial switch procedure, biventricular repair etc.). Serum lactate was higher in the postoperative death group (SMD: 1.18, 95% CI: 0.59–1.77). Lower postoperative N-terminal pro-B-type natriuretic peptide (NT-pro BNP) levels were associated with lower mortality (OR: 0.23, 95% CI: 0.08–0.68) and shorter mechanical ventilation time (OR: 0.40, 95% CI: 0.18–0.90). Higher serum albumin levels were associated with longer hospital stays (OR: 3.12, 95% CI: 1.66–5.84). Significant heterogeneity was found in serum creatinine, B-type natriuretic peptide (BNP), serum lactate, and NT-Pro BNP. Publication bias was detected in some studies.

**Conclusion:**

Serum lactate, NT-Pro BNP, and serum albumin are reliable biomarkers for predicting adverse outcomes in children with CHD after surgery.

**Systematic Review Registration:**

PROSPERO [CRD42024512753].

## 1 Introduction

Structural abnormalities of the heart and/or great vessels present at birth are referred to as congenital heart disease (CHD) (1), with a global incidence of roughly 0.8%–1.2% in live births (2, 3). CHD subtypes span from comparatively uncomplicated and minor lesions, including ventricular septal defect (VSD) and atrial septal defect (ASD), to more intricate and infrequent lesions like hypoplastic left heart syndrome (HLHS), tetralogy of Fallot (ToF), and transposition of the great arteries (TGA) (4). Pharmacological interventions to alleviate symptoms, complications, and conventional surgery or minimally invasive interventional surgery, are the main methods for treating CHD (5, 6). The introduction of cardiopulmonary bypass has rendered the disease manageable (7). Despite significant reductions in mortality achieved through progress in surgery over recent decades, CHD continues to be a primary cause of deaths related to birth defects (2, 8).

Adverse events after surgery in children with CHD (mortality, length of hospital stay, complication rates, etc.) remain a focus concern. In China, the mortality rate of CHD is rising (9). Compared to older children, neonates and infants are more susceptible to adverse events after CHD surgery (10, 11). Amirnovin et al. (10) found that higher perioperative BNP levels were associated with a greater likelihood of adverse outcomes after surgery. Hunt et al. (12) discovered that elevated preoperative von Willebrand factor (VWF) activity was associated with postoperative thrombosis in children with CHD. Gupta et al. (13) confirmed that elevated NT-proBNP may be a useful marker of adverse outcomes after cardiac surgery in children with CHD. To date, several biomarkers have been shown to be associated with adverse outcomes after CHD surgery, but no meta-analysis has combined the data. This article comprehensively searched and analyzed existing studies to explore the value of biomarkers in predicting adverse outcomes in children with CHD after surgery and to lay a theoretical foundation for constructing a more accurate risk prediction model for postoperative outcomes in children with CHD in clinical practice.

## 2 Materials and methods

### 2.1 Literature search

The meta-analysis following PRISMA guidelines (14) and was registered in PROSPERO (CRD42024512753). PubMed, Embase, Cochrane Library, and Web of Science databases for literature searching up to February 2024 to identify English-language literature on the value of biomarkers in predicting postoperative outcomes in children with CHD. Terms followed were included: “congenital heart disease”, “factor”, “randomized controlled trial”, “cohort study”, and “case-control study”. The search strategy for PubMed is as follows: (((((((((((Defect, Congenital Heart [Title/Abstract]) OR (Heart Abnormality [Title/Abstract])) OR (Congenital Heart Defect [Title/Abstract])) OR (Heart, Malformation Of [Title/Abstract])) OR (Malformation Of Heart [Title/Abstract])) OR (Defects, Congenital Heart [Title/Abstract])) OR (Heart Abnormalities [Title/Abstract])) OR (Heart Defect, Congenital [Title/Abstract])) OR (Congenital Heart Disease [Title/Abstract])) OR (Disease, Congenital Heart [Title/Abstract])) OR (Heart Disease, Congenital [Title/Abstract])) OR (Congenital Heart Defects [Title/Abstract]) AND (((((((((Operative Procedures [Title/Abstract]) OR (Procedure, Operative [Title/Abstract])) OR (Surgical Procedure, Operative [Title/Abstract])) OR (Operative Surgical Procedures [Title/Abstract])) OR (Procedure, Operative Surgical [Title/Abstract])) OR (Surgical Procedures [Title/Abstract])) OR (Procedure, Surgical [Title/Abstract])) OR (Operative Surgical Procedure [Title/Abstract])) OR (Ghost Surgery [Title/Abstract])) OR (surgery [Title/Abstract]) AND (factor [Title/Abstract]) AND ((((((randomized controlled trial [All fileds]) OR (randomized controlled trial [All fileds])) OR (RCT [All fileds])) OR (clinical trial [All fileds])) OR (cohort [All fileds])) OR (case-control [All fileds])) OR (clinical study [All fileds]). Furthermore, the reference lists of every qualifying study underwent a manual review. Two researchers, working independently, conducted searches and evaluated the studies that met the inclusion criteria. Any disagreements that arose during the literature search process were resolved through consensus-based discussions.

### 2.2 Inclusion and exclusion criteria

Inclusion criteria: (1) study design was randomized controlled, cohort, or case-control; (2) study subjects were children with CHD; (3) Biomarker data can be extracted: The continuous variable data contains the mean ± standard deviation, and the categorical variable contains the odds ratios (ORs) and the corresponding 95% Conﬁdence Interval (95% CI); (4) at least one of the following outcomes was assessed: mortality, length of hospital stay, complication rates, or infection rates; (5) sufficient data were available to calculate odds ratios (ORs) or standardized mean differences (SMDs). We excluded reviews, letters, editorials, case reports, conference abstracts, unpublished articles, and non-English articles.

### 2.3 Data extraction and quality assessment

Two researchers (SF Zhou. and L Liu) extracted data independently. Any discrepancies were resolved by a third researcher (SF Ma) who made the final decision. First author, year of publication, study period, country, study design, sample size, age, weight, gender, disease type, biomarker, detection time, threshold, and incidence of adverse outcomes were extracted. When continuous variables were reported as medians with ranges or interquartile ranges in the studies, we calculated means ± standard deviations using validated mathematical methods (15, 16). In instances where data was incomplete or not reported in the studies, we reached out to the corresponding authors to request the missing information. Newcastle-Ottawa Scale (NOS) was utilized to evaluate the quality, with scores ranging from 7 to 9 points indicating high-quality research (17). The assessment of quality and evidence level for eligible studies was carried out independently by two researchers. Any disagreements that arose during this process were resolved through open discussions.

### 2.4 Statistical methods

Review Manager version 5.4 was utilized to conduct evidence synthesis. Data were pooled using SMDs for continuous variables and ORs for dichotomous variables. All indicators were reported with 95% confidence intervals (CIs). The chi-squared (*χ*^2^) test (Cochran's Q) and inconsistency index (*I*^2^) were applied for the evaluation of the heterogeneity of each outcome. *χ*^2^ *P* value more than 0.05 or *I*^2^ < 50% indicating non-significant heterogeneity and a fixed-effects model used for data synthesis. Conversely, when *χ*^2^ *P* value less than 0.05 or *I*^2^ ≥ 50%, a random-effects model was used. Moreover, we conducted sensitivity analysis to evaluate how the incorporated studies influenced the pooled findings for outcomes exhibiting substantial heterogeneity. To visually assess potential publication bias for outcomes with three or more included studies, we employed Egger's regression test utilizing the statistical software Stata, version 15.0. For publication bias, statistical significance was indicated by a *P*-value below 0.05.

## 3 Results

### 3.1 Identification of relevant studies

**Figure 1:**
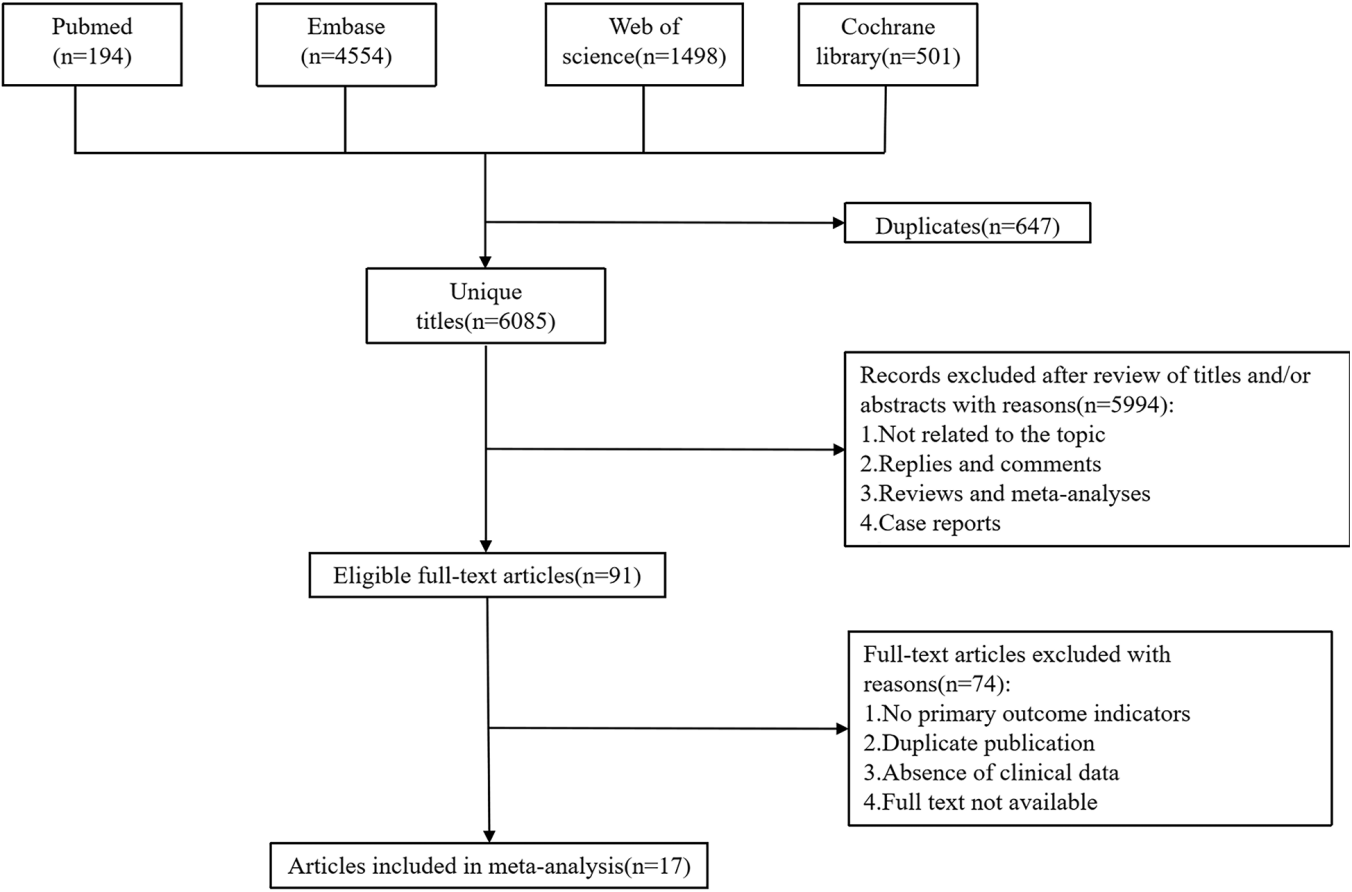
Flowchart onf the systematic search and selection process.

The initial search yielded a total of 6,747 potentially relevant studies. Duplicate studies were eliminated, leaving 6,085 unique records. After reviewing titles, abstracts, and full-text articles against the predefined inclusion and exclusion criteria, 17 studies met the eligibility requirements. These qualified studies encompassed a total of 2,888 children (involved patients younger than 18 years), which were included in the pooled analysis (10, 12, 13, 18–31). The flow chart was shown in Figure 1.

### 3.2 Study characteristics and quality assessment

Among the 17 included studies, 8 were prospective studies and 9 were retrospective studies. Regarding the time of biomarker measurement, 10 studies were preoperative, 6 were postoperative, and 1 was not mentioned (Table 1). Three studies used serum lactate as a biomarker, two used NT-ProBNP, two used PaO2, and the remaining studies used serum creatinine, C1-INH activity, ST2, serum chloride concentration, GH, glycemia, cTOI, NLR, serum albumin, or glucose levels as biomarkers. The observed outcomes varied by study: 7 studies used mortality as an outcome, 4 studies reported the incidence of postoperative AKI, and the remaining studies used thrombosis (coronary thrombosis, arterial thrombosis, deep venous thrombosis, arterial stroke, cerebral sinus thrombosis, shunt thrombosis), prolonged hospitalization (Neonate >46 days, infant >17 days), capillary leak syndrome (the development of generalized noncardiac edema, ascites, pleural effusion, and a weight gain of more than 10%), prolonged PICU length of stay (>3 days), infection rate (positive culture from blood, urine, or wound), prolonged mechanical ventilation time (Neonate >184 h, infant >22 h) as outcome events. The quality evaluation utilizing the NOS scale revealed that 10 of the incorporated studies achieved scores ranging from 7 to 9 points, suggesting good quality and a low risk of bias. The characteristics and quality ratings of the eligible studies are presented in Table 1.

**Table 1:** Baseline characteristics of include studies and methodological assessment.

Authors	Study period	Country	Study design	Patients	Gender		Age	Weight	Type of disease	Biomarker	Detection time	Threshold	Quanlity score	Mode of operation
					Male	Female								
Amirnovin et al. (10)	2005–2009	USA	Prospective cohort	34	24	10	9 ± 8d	3.3 ± 0.4	UVH	Serum lactate	12 h Post-CPB	NA	5	Modified Norwood procedure, arterial switch procedure, biventricular repair
Molina Hazan et al. (18)	1999–2001	Israel	Retrospective cohort	255	140	115	2.7 ± 4.3yr	NA	NA	Serum lactate	At the end of the surgical procedure	NA	6	NA
Hunt et al. (12)	2015–2016	USA	Prospective cohort	133	79	54	NA	4.2 ± 1.4	SV, 2V	Serum lactate	Preoperative	NA	6	NA
Abella et al. (19)	2004–2008	Italy	Case control study	48	24	24	4.6 ± 2mos	NA	TOF, TGA,AS, TA	PaO2	Pre-operative	NA	8	NA
Volovelsky et al. (22)	2016–2017	USA	Prospective cohort	76	42	34	0.9 ± 0.9 years	NA	Cyanotic, non-cyanotic	Serum creatinine	Pre-operative	NA	7	NA
Stiller et al. (23)	NA	Germany	Prospective cohort	27	14	13	NA	NA	VSD, ASD, CAVSD, TAPVD, TOF, TGA, CoA, DOLV	C1-INH activity	Preoperative	NA	8	NA
Parker et al. (24)	2010–2014	Lebanon	Prospective cohort	162	97	65	281 ± 1184.4days	12.1 ± 19.4	NA	ST2	Preoperative	NA	7	NA
Kimura et al. (25)	2013–2017	Japan	Case control study	521	278	243	12.2 ± 4.4months	7.4 ± 4.8	NA	Serum chloride concentration	Preoperative	NA	7	NA
Leite et al. (26)	1994–1996	Brazil	Prospective cohort	36	20	16	8.7 ± 6.5 months	NA	VSD, ASD, PDA, CAVSD, TAPVD, DOLV, TOF, CoA	GH	Fifth postoperative days	NA	6	NA
Lou et al. (20)	2009–2009	China	Case control study	100	68	32	6.7 ± 0.3months	7.1 ± 0.2	SV, 2V	Glycaemia	Pre-operative	NA	7	NA
Aly et al. (27)	NA	USA	Prospective cohort	75	44	31	5.1 ± 0.8 days	3.4 ± 0.6	SV, 2V	cTOI	Preoperative	58%	6	NA
Perez-Piaya et al. (28)	2007–2008	Spain	Prospective cohort	68	21	47	29 ± 38.4months	6.8 ± 11.7	VSD, ASD, TAPVR, PR, AR, PS, CoA, PAIVS, TOF, SV, TGA	NT-ProBNP	Preoperative	3.4	5	NA
Gupta et al. (13)	2015–2017	China	Retrospective cohort	873	304	569	NA	NA	VSD, ASD, TOF, TGA, PAVM, CoA, PAIVS, DOLV, PS	NT-ProBNP	Postoperative	NA	8	NA
Manuel et al. (29)	2011–2017	Brazil	Retrospective cohort	61	24	37	NA	NA	NA	NLR	NA	NLR < 1	8	The bidirectional Glenn procedure
Henry et al. (30)	2007–2013	USA	Case control study	200	120	80	NA	NA	NA	Serum albumin	Pre-operative	2.5	6	NA
Sznycer-Taub 2016	2015–2017	USA	Case control study	93	54	39	7.6 ± 3 days	3.3 ± 0.2	NA	Pao2	The first 48 h	>193	8	NA
Odek et al. (31)	2008–2013	Turkey	Retrospective cohort	126	62	64	17.2 ± 32.3mos	7.9 ± 5.4	VSD, TOF, CAVSD, ASD, ASD + VSD, PDA, PAIVS, VSD + PDA, CoA, TAPVR, VSD + PAPVR, ASD + PAPVR, AL	Glucose levels	Postoperative	NA	8	VSD closure Pulmonary banding TOF repair AVSD repair ASD closure ASD + VSD closure PDA ligation BT-shunt Glenn procedure Fontan procedure VSD closure + PDA ligation Coarctation of aorta repair Subaortic ridge resection TAPVR repair PAPVR repair + VSD closure PAPVR repair + ASD closure Coronary switch Double aortic arch repair, Homograph patch augmentation Extended aortoplasty

UVH, univentricular heart; SV, single ventricle; 2V, two-ventricle repairs; TOF, tetralogy of Fallot; TGA, transposition of the great arteries; AS, aortic stenosis; TA, tricuspid atresia; VSD, ventricular septal defect; ASD, atrial septal defect; CAVSD, complete atrioventricular septal defect; TAPVD, total anomalous pulmonary venous drainage; CoA, coarctation of the aorta; DOLV, double-outlet left ventricle; PDA, patent ductus arteriosus; TAPVR, total anomalous pulmonary venous return; PR, pulmonary regurgitation; AR, aortic regurgitation; PS, pulmonary stenosis; PAIVS, pulmonary atresia with VSDPAVM, pulmonary arterio venous malformation; PAPVR, partial anomalous pulmonary venous return; ALCAPA, anomalous left coronary artery from the pulmonary artery; ST2, suppression of tumorigenicity; GH, neutrophil-lymphocyte ratio; cTOI, cerebral tissue oxygenation index; NLR, neutrophil-lymphocyte ratio.

### 3.4 preliminary study synthesis

#### 3.4.1 The pooled data for continuous variable showed

**Figure 2:**
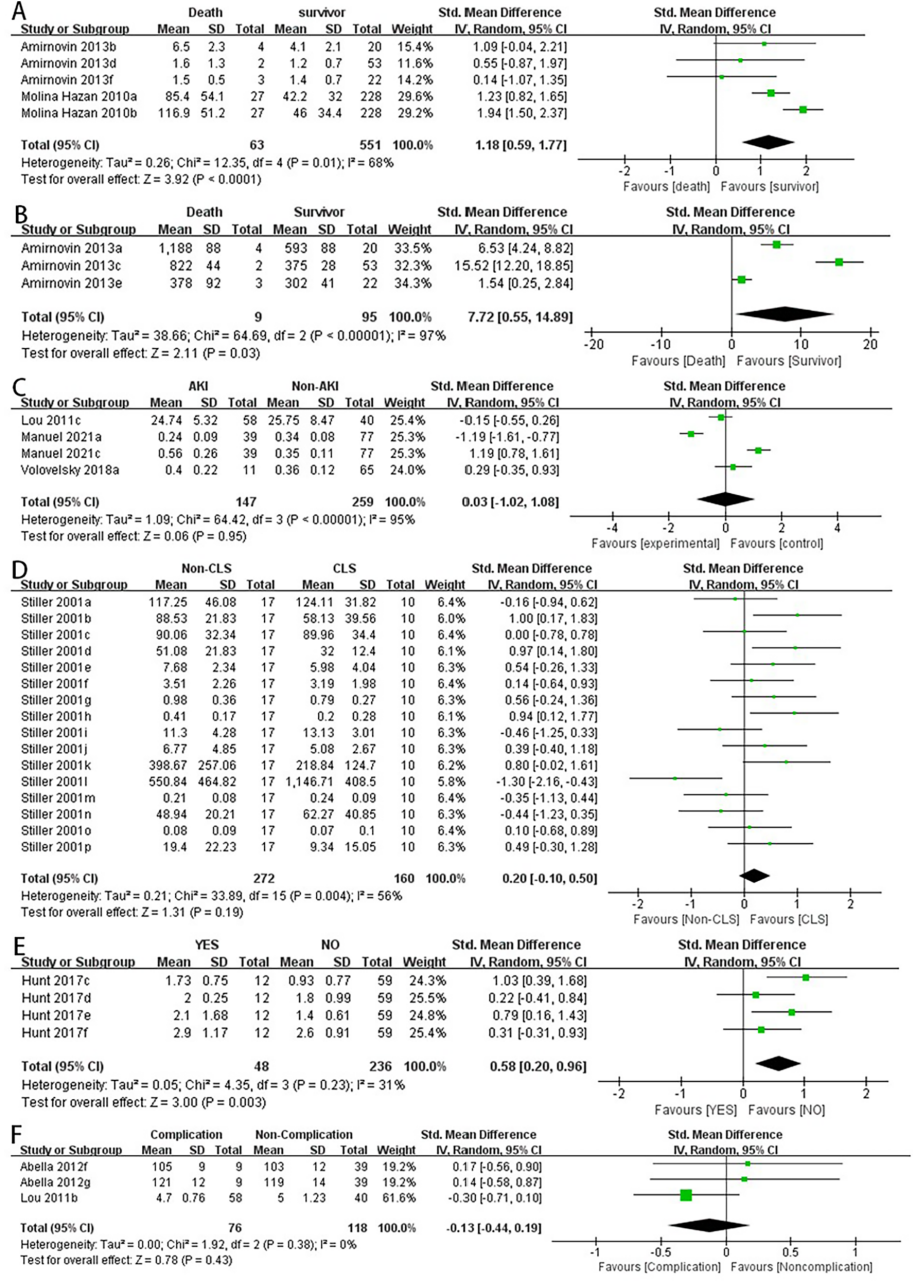
Forest plot of preliminary studies synthesis,lactic acid levels were compared between the dead group and the surviving group **(A)**, BNP levels were compared between the dead group and the surviving group **(B)**, serum creatinine levels were compared between the two groups whether AKI occurred **(C)**, comparison of activation of the serum complement system and the occurrence of CLS after surgery in children with CHD **(D)**, comparison of preoperative VWF activity between the two groups for postoperative thrombosis **(E)**, comparison of blood glucose levels between the two groups for postoperative complications **(F)**.

1.1 A summary analysis of the 95% CIs corresponding to the biomarkers and their outcomes extracted from the included studies showed that the serum lactate level was higher in the postoperative death group compared to the survival group in children with CHD (SMD: 1.18, 95% CI: 0.59–1.77), with significant heterogeneity (*I*^2^ = 68%, *P* < 0.0001) and a random-effects model used (Figure 2A).
1.2 The BNP level was higher in children who died after CHD surgery compared to those who survived (SMD: 7.72, 95% CI: 0.55–14.89), with significant heterogeneity (*I*^2^ = 97%, *P* = 0.03) and a random-effects model used (Figure 2B).
1.3 Preoperative VWF activity was higher in children with CHD who developed thrombosis after surgery compared to those without thrombosis (SMD: 0.58, 95% CI: 0.20–0.96), with a random-effects model used (Figure 2E).
1.4 Serum creatinine levels were not significantly associated with the occurrence of acute kidney injury (AKI) after surgery in children with CHD (SMD: 0.03, 95% CI: −1.02 to 1.08), with significant heterogeneity (*I*^2^ = 95%, *P* = 0.95) (Figure 2C).
1.5 Activation of the serum complement system was not significantly associated with the occurrence of capillary leak syndrome (CLS) after surgery in children with CHD (SMD: 0.20, 95% CI: −0.10 to 0.50) (Figure 2D).
1.6 No statistically significant difference was observed in the blood glucose levels of the two groups, whether they experienced complications after surgery or not (SMD: −0.13, 95% CI: −0.44 to 0.19) (Figure 2F).

#### 3.4.2 The pooled data for categorical variables showed

**Figure 3:**
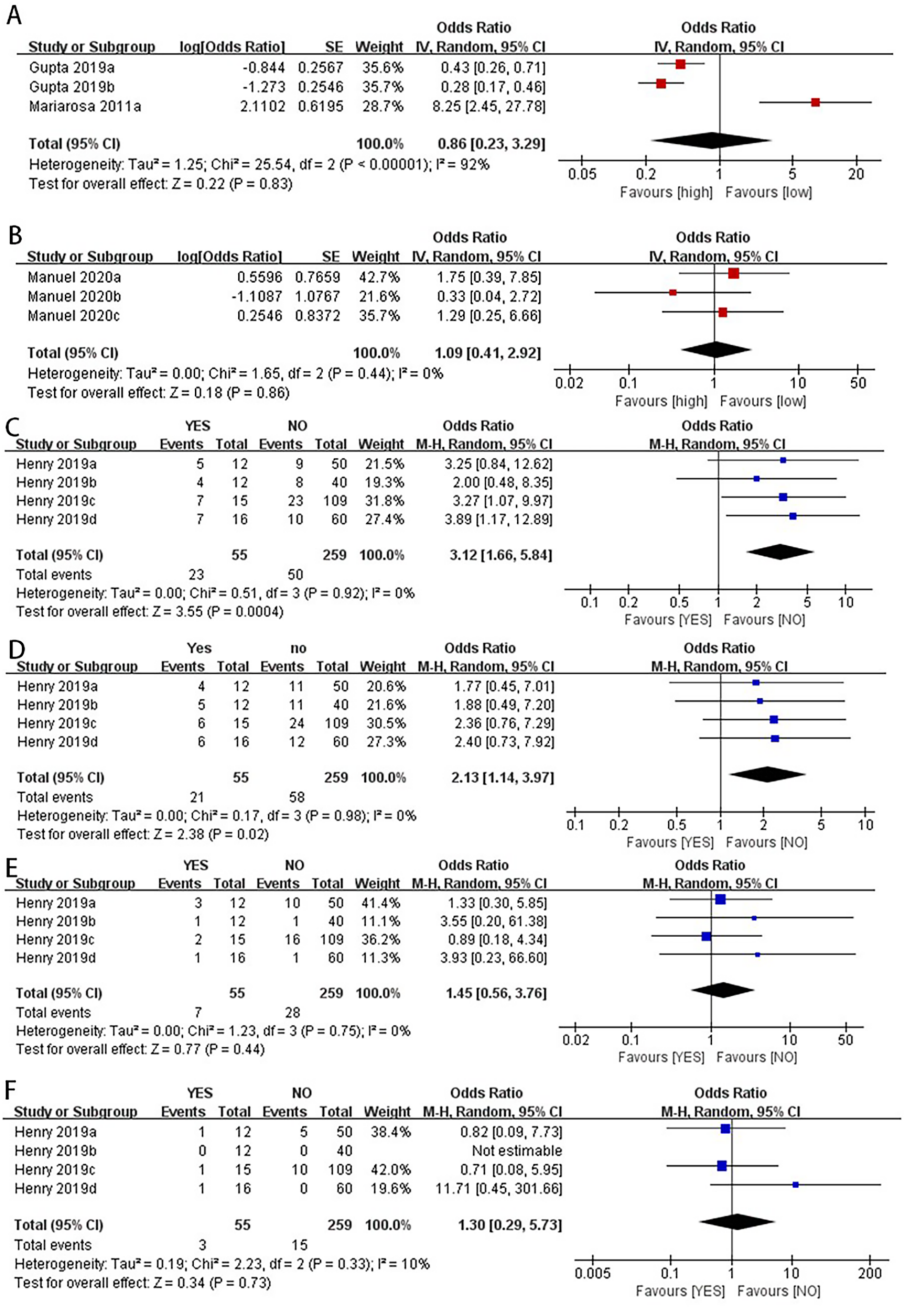
Categorical variables combined data analysis.NT-proBNP levels were not significantly associated with prolonged PICU stay in children after surgery **(A)**. There was no significant difference in postoperative mortality between the high NLR and low NLR groups in children with CHD **(B)** the pooled data for categorical variables showed that lower serum albumin levels were associated with an increased incidence of postoperative hypoalbuminemia in children with CHD **(C)** lower serum albumin levels were associated with longer hospital stays in children with CHD after surgery **(D)** there were no significant differences in postoperative mortality or infection rates between the high serum albumin and low serum albumin groups **(E,F)**.

2.1 Lower serum albumin levels were associated with an increased incidence of postoperative hypoalbuminemia in children with CHD (OR: 3.12, 95% CI: 1.66–5.84) (Figure 3C).
2.2 Lower serum albumin levels were associated with longer hospital stays in children with CHD after surgery (OR: 2.13, 95% CI: 1.14–3.97) (Figure 3D).
2.3 NT-pro BNP levels were not significantly associated with prolonged PICU stay in children after surgery (OR: 0.86, 95% CI: 0.23–3.29) (Figure 3A).
2.4 There was no significant difference in postoperative mortality between the high NLR and low NLR groups in children with CHD (OR: 1.09, 95% CI: 0.41–2.92) (Figure 3B).
2.5 There were no significant differences in postoperative mortality or infection rates between the high serum albumin and low serum albumin groups (OR: 1.45, 95% CI: 0.56–3.76) (Figure 3E) (OR: 1.30, 95% CI: 0.29–5.73) (Figure 3F).

We also statistically pooled studies that used mortality, AKI incidence, and thrombosis as outcome indicators, with details shown in Tables 2–4.

**Table 2:** Data pooled with mortality as an outcome indicator.

Biomarkers	Mortality				
	Study	Metrics	Estimate [95%CI]	*P* value	*I* ^2^
Serum lactate (continuous)	5	SMD	1.18 [0.59, 1.77]	<0.0001	68%
BNP (continuous)	3	SMD	7.72 [0.55, 14.89]	0.03	97%
cTOI (continuous)	2	SMD	−0.59 [−2.03, 0.85]	0.42	91%
NT-ProBNP (classified)	2	OR	0.23 [0.08, 0.68]	0.008	0%
NLR (classified)	3	OR	1.09 [0.41, 2.92]	0.86	0%
Glucose levels (classified)	2	OR	0.33 [0.10, 1.70]	0.06	30%
Serum albumin (classified)	4	OR	1.02 [0.18, 5.94]	0.98	36%

BNP, B-type natriuretic peptide; cTOI, cerebral tissue oxygenation index; NT-Pro BNP, N-terminal pro-brain natriuretic peptide; NLR, neutrophil–lymphocyte ratio; OR, odds ratio; CI, confidence interval.

**Table 3:** Data merging with AKI as an outcome indicator.

Biomarkers	AKI				
	Study	Metrics	Estimate [95%CI]	*P* value	*I* ^2^
Serum creatinine (continuous)	4	SMD	0.03 [−1.02, 1.08]	0.95	95%
Serum chloride concentration (continuous)	2	SMD	0.21 [−0.04, 0.46]	0.1	75%
Serum sodium concentration	2	SMD	0.05 [−0.31, 0.42]	0.78	88%

OR, odds ratio; CI, confidence interval.

**Table 4:** Data merging with thrombosis as an outcome indicator.

Biomarkers	Thrombosis				
	Study	Metrics	Estimate [95%CI]	*P* value	*I* ^2^
Serum lactate (continuous)	2	SMD	0.70 [−0.68, 2.08]	0.32	90%
VWF (continuous)	4	SMD	0.58 [0.20, 0.96]	0.003	31%
ADAMTS−13 (continuous)	2	SMD	−0.46 [−1.04, 0.11]	0.12	41%

VWF, von Willebrand factor; OR, odds ratio; CI, confidence interval.

### 3.5 Sensitivity analysis

**Figure 4:**
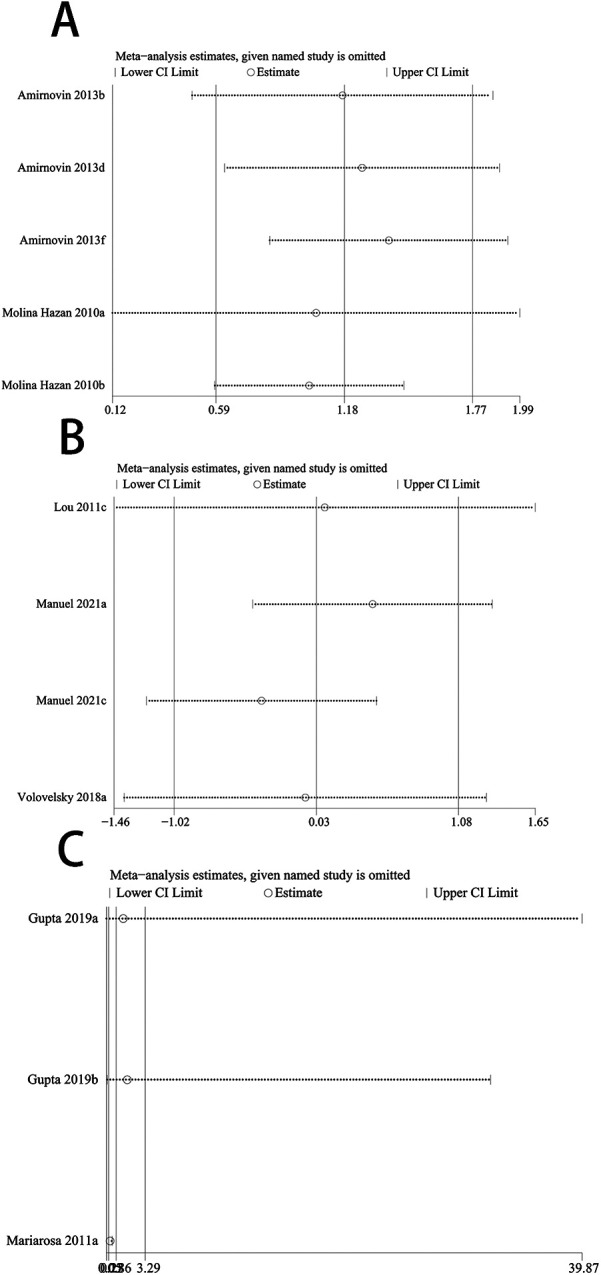
Forest plot of re-synthesis after eliminating one study identified by sensitivity analysis. Sensitivity analysis was performed for studies with *I*^2^ greater than 50%, *I*^2^ = 68% **(A)** *I*^2^ = 95% **(B)** both studies showed stability after the analysis was completed, When we excluded the data reported by Mariarosa et al. in 2011, the new OR value changed from non-significant to significant **(C)**.

The sensitivity analysis aimed to assess the robustness of the findings by altering certain assumptions and provide an initial exploration into the potential sources contributing to the heterogeneity observed across the included studies. Our sensitivity analysis showed that when we excluded the data reported by Mariarosa et al. in 2011 (28), the new OR value changed from non-significant to significant (OR: 0.35, 95% CI: 0.23–0.53) (Figure 4C).

### 3.6 Publication bias assessment

Considering the limited number of included studies, Egger's test was used to assess publication bias for serum creatinine, glycemia, BNP, serum lactate, VWF, serum albumin, NLR, NT-ProBNP, and serum complement system activation. The results showed significant publication bias for glycemia (*P* = 0.02), VWF (*P* = 0.009), and NLR (*P* = 0.04), as well as publication bias for serum albumin in predicting postoperative mechanical ventilation time (*P* = 0.04) and mortality (*P* = 0.04); there was no significant publication bias for serum lactate (*P* = 0.21) and BNP (*P* = 0.10) in predicting postoperative mortality, serum creatinine (*P* = 0.18) in predicting postoperative AKI incidence, serum complement system activation (*P* = 0.82) in predicting postoperative CLS incidence, and serum albumin in predicting postoperative infection rate (*P* = 0.09), total hospital stay (*P* = 0.16), and prolonged PICU stay (*P* = 0.14).

## 4 Discussion

Congenital heart disease occur during cardiac development and are present at birth (32)and is a major global health problem (33). Although great progress has been made in the treatment of CHD in recent years, it remains a significant cause of childhood mortality (34). Among children undergoing surgery for CHD, 10% to 20% are unexpectedly readmitted within 30 days, and 4% of surgeries result in death (35–38). Repeated hospitalizations of children place an emotional and economic burden on patients’ families and strain the healthcare system (39). There is strong interest in identifying early markers to predict impending major adverse events following surgical repair of CHD defects, driven by the significant mortality risk associated with most such surgeries (19, 40, 41). Currently, little attention is paid to the prediction of readmission, mortality, or postoperative complications in children after congenital heart surgery, especially the evaluation of biomarkers compared to standalone clinical models (13).

Our study found that the serum lactate level was higher in the postoperative death group compared to the survival group in children with CHD after surgery (SMD: 1.18, 95% CI: 0.59–1.77). The end-product of glycolysis, pyruvate, is converted into serum lactate. In the presence of oxygen, lactate is converted back to pyruvate through mitochondrial processes. However, under anaerobic conditions, cellular lactate concentrations increase, resulting in elevated serum lactate levels. Therefore, hyperlactatemia may reflect tissue oxygen debt, and serum lactate levels can also serve as a clinical indicator of tissue perfusion and systemic oxygen delivery after CHD surgery (42). In response to increased pressure overload, volume expansion, and myocardial wall stress, ventricular myocytes synthesize and secrete NT-proBNP into the bloodstream (43). NT-proBNP has recently emerged as a potential prognostic marker for early postoperative outcomes in pediatric and congenital cardiac surgery (44–46). Nevertheless, inconsistencies exist regarding the findings. For instance, Qu et al. demonstrated NT-proBNP levels 1 h postoperatively strongly predicted prolonged mechanical ventilation, intensive care unit (ICU) stay, and inotropic therapy requirement (13). Our study found that lower postoperative NT-proBNP levels were associated with lower mortality (OR: 0.23, 95% CI: 0.08–0.68) and shorter mechanical ventilation times (OR: 0.40, 95% CI: 0.18–0.90) in children with CHD after surgery. Hypoalbuminemia caused by decreased serum albumin levels can develop through a combination of decreased synthesis, increased degradation, and the dilutional effect of resuscitation (30); our study found that children with higher serum albumin levels had longer hospital stays (random-effects model, OR: 3.12, 95% CI: 1.66–5.84). For infants with preoperative hypoalbuminemia, treating and/or preventing potential protein malnutrition or heart failure, rather than supplementing albumin, may have a greater impact on improving postoperative outcomes.

In addition, our study found that the BNP level was higher in the postoperative death group compared to the survival group in children with CHD (SMD: 7.72, 95% CI: 0.55–14.89). BNP, a cardiac hormone with diuretic, natriuretic, and vasodilatory properties, is primarily secreted by the ventricles in response to volume expansion and pressure overload. BNP levels are elevated in various cardiovascular diseases, such as heart failure and myocardial infarction (47). Although BNP serves as a biomarker for diagnosing, risk stratifying, and managing heart failure in adults, its applications in neonates and infants undergoing surgical repair or palliation of congenital heart defects remain unestablished (10). VWF, a large multimeric glycoprotein, mediates platelet-platelet and platelet-subendothelial adhesion. After release from activated platelets and endothelial cells, VWF exists primarily as large, highly reactive multimers (12). Our analysis found that preoperative VWF activity was higher in children who developed thrombosis after surgery compared to those without thrombosis (SMD: 0.58, 95% CI: 0.20–0.96).

However, our meta-analysis also has certain limitations, we focused on the short-term predictive value of biomarkers after CHD surgery, and the data we could collect were too small to conduct a meta-analysis on the long-term prognosis (cardiac output, neurodevelopment eta.), which also suggests that a larger number of studies are needed to provide original data. The type of surgery received by patients and albumin was used preoperatively as resuscitation fluid may affect the predictive value of biomarkers. However, the data provided by the included literature were limited, and there was no way to conduct subgroup analysis, which led to a certain risk of bias in the study results, which needs to be solved by further research. Despite removing a highly sensitive study and re-synthesizing the data, moderate heterogeneity persisted even with a random-effects model, potentially compromising the robustness of our conclusions. Secondly, some of the included studies were retrospective, with poor control of confounding factors, resulting in lower quality of some studies. Although this analysis included a wide range of biomarkers, the detailed data for each indicator were not sufficient, and some studies had high heterogeneity and publication bias. However, our study is the first and largest to explore the value of biomarkers in predicting adverse outcomes in children with CHD after surgery from an evidence-based medicine perspective. The analysis was relatively comprehensive and can provide guidance for future clinical treatment and a theoretical basis for constructing clinical risk prediction models.

## 5 Conclusion

Serum lactate, NT-proBNP, and serum albumin are valuable biomarkers for predicting adverse outcomes in children with CHD, as higher serum lactate and NT-proBNP levels and lower serum albumin levels indicate poorer postoperative prognosis in children with CHD. Nonetheless, further investigation is warranted to elucidate the relationship between these biomarkers and CHD prognosis.

## Data Availability

The original contributions presented in the study are included in the article/Supplementary Material, further inquiries can be directed to the corresponding author.
